# Supplementary material for: Executive function and the continued influence of misinformation: A latent-variable analysis
Source: PLoS One. 2023 Apr 5;18(4):e0283951. doi: 10.1371/journal.pone.0283951 (PMC10075451; doi:10.1371/journal.pone.0283951)
Supplement: Attachment — Submitted filename: Response to Reviewers.docx [file pone.0283951.s001.docx]

**Response to Reviewers - PONE-D-22-17797**

**Editor**

1. I was a bit confused about the presentation of the results of Sanderson et al. vs. Brydges et al. It is not really clear what could explain their divergent results, and how the present study can help us further understand the relation between working memory and CIE. Please try to make a more straight point regarding the current state of the literature and what could explain these divergent findings, and how the present study advances the state of this debate.

RESPONSE: Thank you for letting us know that this was confusing. We have added a potential explanation for the conflicting results of Brydges et al. (2018) and Sanderson et al. (2021), in order to provide clarification.

IN-TEXT-EDIT EXAMPLE (p.25): “The current results expand upon the findings of Brydges et al. (17), who demonstrated a predictive relationship between working-memory capacity and CIE susceptibility. However, it should be noted that Sanderson et al. (18) failed to replicate Brydges et al.’s findings; they also conducted a reanalysis of Brydges et al.’s data that yielded a non-significant relationship between the CIE and working-memory-capacity variables. Yet, these contradictory results may have been due to the working-memory capacity measures used by Brydges et al. and Sanderson et al.; that is, these measures may not have tapped into working-memory updating sufficiently to produce a reliable effect with CIE measures. Nonetheless, Brydges and colleagues speculated that their results could be evidence for the mental-model-updating account of the CIE, as the updating mechanism central to this account arguably relies on working memory.”

2. As also noticed by Reviewer 1, there seems to be a conflation between updating and working memory capacity in the paper. Although R1 points it out only as a matter of measurement, I also noticed that the authors use these terms quite interchangeably, creating a bit of confusion, specially in describing their prior work (Sanderson et al. vs. Brydges et al.). Please be consistent in using the term updating when referring to scores derived from updating tasks, and working memory capacity to refer to scores of tasks that do not include updating (or that have a mix of both types of tasks). If the authors do not believe this distinction is relevant, I'd suggest that they are upfront about this in the intro (following the suggestions of R1) and opt for only one term throughout.

RESPONSE: We acknowledge that the term updating was sometimes misplaced and have rectified this.

3. For the stroop task the authors say at some point that they had a congruent condition. Yet, it seems to me that the implemented condition would be more reasonably classified as a neutral baseline since no semantic information was provided (colored squares?).

RESPONSE: We now only refer to the condition as a control.

4. I commend the authors for sharing their data, but as also noticed by R1, only processed data was provided. I wonder if the authors could also provide raw tasks scores. This would allow for more freedom for future studies to explore different task scoring methods. Please also consider adding a codebook (i.e., meta-data file explaining the variables) to the project page.

RESPONSE: Raw data have been added to the study’s OSF page.

5. I wondered a bit about the choice of building difference scores to create the CIE latent variable, and the rather arbitrary choice of which scores to subtract from which scores. I wonder why the authors did not create a CIE factor from a latent difference score? One could create a latent variable that reflects performance in all eight indicators and then one CIE factor that contains only the additional variance from the four correction conditions. Wouldn't that be less arbitrary? Here also providing the raw CIE task scores would be helpful, since at the moment, only the difference scores selected by the authors are available at the OSF and alternative model implementations are not possible.

RESPONSE: We appreciate the point being made here, however, we feel the need to point out that our CIE-difference-score pairings were not arbitrary per se; rather, the correction and non-correction scores were rank ordered first, then we paired the highest through to the lowest scores. Furthermore, the way in which difference scores were paired made no difference to the overall CIE mean, in the end; in fact, it only impacted the estimate for internal-consistency reliability. Therefore, the results of the study would not change based on the CIE-difference-score pairings. That being said, we tested the model being suggested, which, if we are not mistaken, is a bifactor model with a nested CIE factor; results showed that model fit that was, overall, unacceptable, χ^2^(16) = 26.19, *p* = .051, *CFI* = .941, *TLI* = .896, *SRMR* = .041, *RMSEA* = .050 (90% *CI* [.000, .083]). Thus, using the suggested CIE model in further analyses would arguably be inappropriate. However, the raw data now provided on the OSF will allow for anyone to test alternative models.

6. I wondered about the memory measures from the CIE tasks. Could that data also be provided in the OSF? Is there a reason why at least the descriptives are not provided? Could this data provide any additional information?

RESPONSE: We have now provided raw data for the event-related-memory subtask of the CIE task on the OSF page. Furthermore, we ran a correlational analysis on the mean of the event-related memory questions and the CIE scores, finding a significant negative correlation (*r* = -.460, *p* < .001). However, we also found a KMO value of .56 for the event-related memory questions, meaning they were unsuitable for use in further SEM analyses (Kaiser & Rice, 1974); further, the internal-consistency was incalculable due to negative covariances between some of the event-related-memory questions. Nevertheless, the significant negative correlation suggests that engagement with the materials, and thus episodic memory for CIE-task events, played some role in determining peoples’ CIE score. Therefore, we have added the above correlation results to the manuscript and updated the discussion to address this finding.

IN-TEXT-EDIT EXAMPLE (p.28): “Furthermore, Sanderson et al. (18) suggested that greater fidelity of the episodic memory representation of event reports predicted lower susceptibility to the CIE. Similarly, our analyses demonstrated a negative correlation between event-related memory and CIE susceptibility, though poor psychometric properties of our event-related-memory measure prevented further analysis with structural equation modelling. It may be worth further investigating whether episodic-memory ability more generally predicts CIE susceptibility, using more general measures of episodic memory. Moreover, it is possible that episodic-memory abilities could interact with the relationship between EF and the CIE. Indeed, those with better episodic memory may generate higher-fidelity mental models, which may in turn be easier to update. Future investigations could thus seek to assess how the relationship between EF and CIE susceptibility may change across the spectrum of episodic-memory ability, or in other words, determine if episodic memory moderates the EF-CIE relationship.”

Reference:

Kaiser, H. F., & Rice, J. (1974). Little jiffy, mark IV. *Educational and Psychological Measurement*, *34*(1), 111-117. https://doi.org/10.1177/001316447403400115

**Reviewer 1**

The presented manuscript discussed if individual differences in the continued influence effect (CIE) of misinformation can be explained by executive functions. The theoretical introduction is clear, and the tested hypothesis are coherently derived from previous literature. The collected measures are well described and mostly adequate to test the hypothesis (see my comments below for specific critique points). The analysis is also clearly written and fitting for testing the hypothesis, although some more details could have been provided in the manuscript (see my detailed comments). The results are clearly presented and easy to comprehend. Likewise, the discussion summarizes the results well and integrates the presented findings well into the already existing literature. All in all, I think this is an interesting and well written manuscript that will make an important contribution for understanding the continued influence effect and individual differences therein. Yet, there are still some issues that I would like to see addressed prior to publication. These are outlined below.

1. Finding psychometrically good and valid indicators of executive functions is difficult and all in all I think the authors have made a balanced selection for the different executive functions. The only issue I see is that for updating only average performance in working memory tasks involving updating was used. This measure likely conflates individual differences in storage processes of working memory with executive processes like updating. Specifically, previous research has shown that updating tasks are equally good indicators of working memory capacity as simple binding tasks that do not require any updating and removal of outdated information (Wilhelm et al., 2013). This indicates that updating performance likely mainly measures individual differences in more basic WM processes not related to updating. This was further validated in a recent study of my own (Frischkorn et al., 2022). This is not to say, that I want the authors to cite my work, but I rather want to point out that a more nuanced discussion of their updating measurement is required, especially with respect to the discussion and implications of their results. It could be that primarily basic working memory processes such as storage & maintenance are related to individual differences in CIE, because the current measure does not sufficiently separate processes specific to updating from these basic processes.

RESPONSE: Thank you for this thoughtful point, and we agree that this is a relevant and important nuance to include in our discussion. Consequently, we have added a paragraph to the Discussion that addresses the issues surrounding the measurement of updating.

IN-TEXT-EDIT EXAMPLE (p.26): “However, it should be acknowledged that the updating measures used in the current study may not have been pure measures of updating; that is, other aspects of working memory may have also been measured. Indeed, while the Keep-track and N-Back tasks are commonly used as updating measures (e.g., 19,38,62-64), some evidence suggests that these tasks also measure more basic storage and maintenance operations in working memory (e.g., 65-66). Therefore, despite our attempts to isolate updating-specific variance with a latent-variable approach, we cannot definitively conclude from our results that updating was the only working-memory process predicting CIE susceptibility.”

2. I thank the authors for making their materials and data available. I was able to reproduce all the results and I think this is excellent for the promotion of open science. Yet, in reproducing the results I noted that some data analytical choices were not sufficiently reported in the manuscript.

a. How was the scaling of latent variables achieved? Did the authors fix the loading of one of the indicators to one (i.e., normalization), or did they fix the variance of the latent variables to one (i.e., standardization). This is a minor point, but for a complete description of the analysis please add this information

RESPONSE: We used the standardisation method. This information has been added to the manuscript.

IN-TEXT-EDIT EXAMPLE (p.16): “All CFAs and SEMs were run in AMOS 27 (50) using maximum-likelihood estimation. We used standardisation as the scaling method for our latent variables. Our point-estimate CIs were estimated using bootstrapping (2,000 samples). Model fit was determined using the following criteria from Schweizer (51): comparative fit index (CFI) ≥ .950; Tucker-Lewis index (TLI) ≥ .950; standardised root mean-square residual (SRMR) < .08; root mean square error of approximation (RMSEA) < .06 (including 90% CIs).”

b. It seems that the authors have standardized the observed variables prior to estimating SEM. I understand this decision given that variances vary considerably between measures. This is not apparent in the current version of the manuscript. Again, I do not think that this is a serious problem, but in the interest of transparency I suggest adding this information and discussing the implications of performing SEM based on correlation matrices instead of covariance matrices

RESPONSE: Apologies if we are misunderstanding, but all the task scores reported in the descriptives table are unstandardised; that is, all response-time-task scores are reported in milliseconds and all task scores based on proportion correct are reported as decimals. These same scoring scales were used for the observed variables in our SEMs. As for the CIE task, we simply transformed the scores from a 0 – 10 scale (from the 11-point Likert scale in our CIE task) to a 0 – 1 scale. If, in fact, the reviewer is referring to using standardised beta-weights and correlations in our SEMs (rather than unstandardised beta-weights and covariances), we believe that the standardised estimates are easier to interpret and more informative.

3. When playing around with the data a bit, I noted that the main problem for the correlated EF model causing its bad fit was the cross loading of the Trail-Making Task on the updating factor. This is the same cross-loading later implemented in the model including just shifting and updating. Why did the authors choose to not include this cross loading in the model with all three executive functions given that this would have achieved a good fit? This would then offer the possibility to include inhibition in their analysis as a predictor of individual differences in the CIE. I know that there were additional reasons to drop inhibition, but maybe including the cross-loading can mitigate problems with including inhibition in the SEM analysis.

RESPONSE: We have now added an analysis of the alternative correlated-three-factor model to the Online Supplement and have referred to this analysis in the manuscript. However, while we appreciate the point being made here, we believe that the inhibition factor is unsuitable for use in further analyses because: 1) the inhibition factor had a near-perfect correlation with the updating factor; 2) the updating factor had significantly larger factor loadings than the inhibition factor; and 3) the upper confidence intervals for the inhibition-factors correlations with the updating and shifting factors exceeded 1. In combination, these three points suggest that inhibition was not being validly measured, further suggesting that analyses performed with the inhibition factor would likely produce invalid results.

IN-TEXT-EDIT EXAMPLE (p.21): “In our CFA of a correlated two-factor model defined by updating and shifting latent variables, model fit was found to be unacceptable, χ2(8) = 22.90, p = .003, CFI = .919, TLI = .847, SRMR = .060, RMSEA = .086 (90% CI [.046, .128]); however, when we allowed a cross-loading for the Trails task, model fit was excellent, χ2(7) = 4.69, p = .698, CFI = 1.000, TLI = 1.027, SRMR = .020, RMSEA = .000 (90% CI [.000, .059]). Indeed, the Trails task has been found to yield cross-loadings onto updating and shifting factors previously.(e.g., 58) As can be seen in Figure 2, all factor loadings were positive for both latent variables, and all loadings were significant (p < .001). Further, while the latent variable correlation was significant and large in the standard correlated two-factor model (r = .55, 95% CI = [.27,.76], p < .001), it was non-significant with the cross-loading included (r = .14, 95% CI [ -.08, .35], p = .208). Note that including the Trails cross-loading in the correlated three-factor model also produced acceptable model fit; however, due to issues highlighted for the inhibition factor in the above paragraph, the correlated two-factor model was preferred. For transparency, results of analyses with the correlated three-factor model have been provided in the Online Supplement.”

4. Related to my previous point, the authors could have dropped the flanker task as an indicator of inhibition and constrained the loading of Stroop & go-no-go to be equal for the inhibition factors. That way the inhibition factor would be still locally identified, and the authors could present results with respect to relationship of inhibition with individual differences in the CIE.

RESPONSE: We understand the point being made here; however, we feel that there are two main issues with what the reviewer proposes. Firstly, if the Flanker task was dropped from the inhibition factor, the inhibition factor would still demonstrate the psychometric weaknesses highlighted in our response to the reviewer’s third point. Secondly, a latent variable typically requires at least three observed measures to be stable (e.g., Marsh & Hau, 2007). Thus, any results derived from the inhibition factor would likely be unreliable and invalid. However, we have added the proposed analysis to the Online Supplement, as it will allow for easy comparison of the results.

Reference:

Marsh, H. W., & Hau, K. T. (2007). Applications of latent-variable models in educational psychology: The need for methodological-substantive synergies. *Contemporary Educational Psychology*, *32*(1), 151-170. https://doi.org/10.1016/j.cedpsych.2006.10.008

5. I was surprised to see that the reliability of the differences scores for inhibition was so good, especially for the Stroop and flanker task. Could the authors share the full data for estimating these reliabilities and provide more information how reliability was estimated. I am familiar with omega in the context of CFA but computing it for item indicators is less common. I assume that the authors followed the method suggested by Hayes & Coutts (2020), but a more explicit description of the procedure would aid the reader in following the reliability estimation. Given the number of trials per condition (48 or 60) and results from previous research (for a summary see von Bastian et al., 2020) I would like to see some more information regarding these estimates to really trust them.

RESPONSE: Since submitting the manuscript, we have realised that reliability was not calculated correctly for measures with difference scores (i.e., Flanker, Stroop, Category-Switch, and Number-Letter tasks). Specifically, in the submitted manuscript, we calculated reliability for difference scores by simply calculating McDonald’s ω for the experimental and control condition scores combined. However, we should have used the formula for calculating difference-score reliability proposed by Furr and Bacharach (2008, p.132). We have now corrected the reliability estimates for the relevant tasks in the manuscript (p.18), based on Furr and Bacharach’s (2008) formula. Furthermore, we have now provided raw data on the OSF page.

Reference:

Furr, R. M., & Bacharach, V. R. (2008). *Psychometrics: An Introduction*. SAGE.

6. I am not entirely convinced that the additional exploratory factor analysis adds to the robustness of the results. I agree that SEM estimates are unstable with smaller sample sizes, especially if factor reliabilities are low. But the same problem applies to estimates for factor analysis that rely on correlation/covariance estimates. These are also not stable in smaller sample sizes and when correlations are low (Schönbrodt & Perugini, 2013). Therefore, the same critique could be used for the results of the exploratory factor analysis. Maybe I am missing a point here, in this case please elaborate why you think that EFA results should be more robust than the SEM results.

RESPONSE: We can certainly understand the point being made here; however, we take a different point of view. Specifically, for a non-disattenuated correlation of .20, Schönbrodt and Perugini (2013) recommended a sample size of approximately 240 to help obtain stable estimates. For a latent variable correlation of .20, Kretzschmar and Gignac (2019) recommended a sample size of approximately 490 to obtain stable estimates. Given our sample size, the magnitude of the key effect, and the fact that factor loadings derived from an unrestricted factor analysis are not disattenuated, we believe the results from the unrestricted factor analysis help reinforce the conclusions of the manuscript, and thus, we would prefer to keep those results in the manuscript.

References:

Kretzschmar, A., & Gignac, G. E. (2019). At what sample size do latent variable correlations stabilize?. *Journal of Research in Personality*, *80*, 17-22. https://doi.org/10.1016/j.jrp.2019.03.007

Reviewer References:

Frischkorn, G. T., von Bastian, C. C., Souza, A. S., & Oberauer, K. (2022). Individual differences in updating are not related to reasoning ability and working memory capacity. *Journal of Experimental Psychology: General*. https://doi.org/10.1037/xge0001141

Hayes, A. F., & Coutts, J. J. (2020). Use omega rather than Cronbach’s alpha for estimating reliability. But…. *Communication Methods and Measures, 14*(1), 1–24. https://doi.org/10.1080/19312458.2020.1718629

Schönbrodt, F. D., & Perugini, M. (2013). At what sample size do correlations stabilize? *Journal of Research in Personality, 47*(5), 609–612. https://doi.org/10/f496x4

von Bastian, C. C., Blais, C., Brewer, G. A., Gyurkovics, M., Hedge, C., Kałamała, P., Meier, M. E., Oberauer, K., Rey-Mermet, A., Rouder, J. N., Souza, A. S., Bartsch, L. M., Conway, A. R. A., Draheim, C., Engle, R. W., Friedman, N. P., Frischkorn, G. T., Gustavson, D. E., Koch, I., … Wiemers, E. A. (2020). Advancing the understanding of individual differences in attentional control: Theoretical, methodological, and analytical considerations. *PsyArXiv*. https://doi.org/10.31234/osf.io/x3b9k

Wilhelm, O., Hildebrandt, A., & Oberauer, K. (2013). What is working memory capacity, and how can we measure it? *Frontiers in Psychology, 4*, 433. https://doi.org/10/gd3vs7

**Reviewer 2**

This is a very interesting paper and I enjoyed reading it. It’s great to see a systematic examination of the relationship between EF/WM and the CIE that draws on contemporary models of WM such as Miyake’s account. The paper is nicely structured and well written, and the study design and analysis are appropriate for addressing the hypothesis/predictions. I don’t think there is a great deal that needs be to done to revise the paper – the comments that follow raise a few issues that I think can be dealt with relatively little effort. I’m cognisant of the fact that there’s not a lot of space available to add more content.

1. The relationship between the overview of the two models and the hypothesis and predictions felt a little loose. The way I read the introduction felt like it was setting up the study to play off the updating and inhibition accounts. Indeed, the Discussion suggests that the findings favour the former. Yet the hypothesis and its associated predictions don’t make any mention of a test of the claims of the models. It would be nice to see a stronger link forged.

RESPONSE: We agree that there is some disconnect between the rationale built in the Introduction and the hypothesis and predictions. Therefore, we have added to the Introduction more clarification of the study’s exploratory nature; note that we believe the study to be exploratory due to the limited nature of previous individual-differences research on the CIE. Furthermore, we have added clarification in the hypothesis paragraph about the theoretical implications of specific results.

IN-TEXT-EDIT EXAMPLE (p.6): “As the current study is exploratory, due to the limited individual-differences evidence for CIE theoretical accounts (17, 27), we simply hypothesised that greater EF ability would predict lower CIE susceptibility. As such, we predicted that there would be: (i) negative observed-score correlations between one or more of the EF measures and CIE measure, and (ii) a significant negative β weight between one or more of the EF-subcomponent latent variables and the CIE latent variable. However, note that finding such relationships for updating and inhibition measures would provide some evidence for the mental-model-updating and selective-retrieval accounts of the CIE, respectively.”

2. I found it a bit odd that the N-back tasks were described as measures of updating without additional justification. Admittedly there is some face validity for thinking this might be the case but I think it needs to be argued for – especially in light of the debate over what the N-back actually measures and whether it is a WM task at all.

Redick, T. S., & Lindsey, D. R. B. (2013) https://doi.org/10.3758/s13423-013-0453-9

Frost, A., Moussaoui, S., Kaur, J., Aziz, S., Fukuda, K., & Niemeier, M. (2021). https://doi.org/10.1016/j.actpsy.2021.103398

Kane, M. J., Conway, A. R. A., Miura, T. K., & Colflesh, G. J. H. (2007). https://doi.org/10.1037/0278-7393.33.3.615

It would be useful to provide some justification for using the N-back tasks this way. This is especially important given the fact the updating latent variables in the various analyses are strongly (mostly?) ‘driven’ by the N-back measures. If the validity of the N-backs as updating measures is in question then it does raise the questions as to what exactly it is that is explaining variance in the CIE.

RESPONSE: We thank the Reviewer for this comment. In response, and also based on Reviewer 1’s first point, we have added a discussion of our updating measures’ limitations to the Discussion section. While we acknowledge that the subject of what the N-Back task measures is psychometrically contentious, we don’t think that using the N-Back task as an updating, or even general working-memory, measure is entirely unjustifiable. Indeed, Reviewer 1 referenced studies showing evidence that the N-Back task at least measures basic aspects of working memory, as well as updating to a potentially lesser degree (e.g., Frischkorn et al., 2022; Wilhelm et al., 2013); further, the N-Back task is commonly utilised as a measure of updating (e.g., Colzato et al., 2013; Friedman et al., 2008; Laine et al., 2018). Thus, we believe that our additions to the Discussion section cover this potential limitation.

References:

Colzato, L. S., Jongkees, B. J., Sellaro, R., & Hommel, B. (2013). Working memory reloaded: tyrosine repletes updating in the N-back task. *Frontiers in Behavioral Neuroscience*, *7*, 200. https://doi.org/10.3389/fnbeh.2013.00200

Friedman, N. P., Miyake, A., Young, S. E., DeFries, J. C., Corley, R. P., & Hewitt, J. K. (2008). Individual differences in executive functions are almost entirely genetic in origin. *Journal of Experimental Psychology: General*, *137*(2), 201-225. https://doi.org/10.1037/0096-3445.137.2.201

Frischkorn, G. T., Von Bastian, C. C., Souza, A. S., & Oberauer, K. (2022). Individual differences in updating are not related to reasoning ability and working memory capacity. *Journal of Experimental Psychology: General*, *151*(6), 1341-1357. https://doi.org/10.1037/xge0001141

Laine, M., Fellman, D., Waris, O., & Nyman, T. J. (2018). The early effects of external and internal strategies on working memory updating training. *Scientific Reports*, *8*(1), 1-12. https://doi.org/10.1038/s41598-018-22396-5

Wilhelm, O., Hildebrandt, A., & Oberauer, K. (2013). What is working memory capacity, and how can we measure it?. *Frontiers in Psychology*, *4*, 433. https://doi.org/10.3389/fpsyg.2013.00433

3. Although it’s clear that the CIE has been used in individual differences (ID) research before I think there is a question as to whether it is an unproblematic ID measure. The paradigm that is used to generate the CIE measure used in the study is essentially an experimental one (à la (Johnson & Seifert, 1994) where the focus is on creating a task that is best distinguishes different experimental conditions/groups (accentuates between group variation) rather than one that preserves inter-individual variation. Thus, I wonder whether it might suffer from the same kinds of problems other experimental measures (e.g., ‘classic’ task switching and even the Stroop – see Rouder & Haaf, 2019) face when imported into ID research (typically an attenuation of covariance between the ID measures – often to the point where correlations etc are not statistically significant. This in turn can have all sorts of consequences for the likes of CFA, SEM and so on). If this is the case it is unclear to me how it might be influencing the relationships explored in the current study. Might a better ID CIE measure result in a different pattern of results on the SEM? Obviously these questions are beyond the scope of a revision of the paper – but I’d suggest that a brief discussion of the suitability of CIE measure for ID research would be helpful and assure the reader that limitations of the measure don’t mean we might be missing something important.

Hedge, C., Powell, G., & Sumner, P. (2018). https://doi.org/10.3758/s13428-017-0935-1

Rouder, J., Kumar, A., & Haaf, J. M. (2019). https://doi.org/10.31234/osf.io/3cjr5

Rouder, J. N., & Haaf, J. M. (2019). https://doi.org/10.3758/s13423-018-1558-y

RESPONSE: The point being made here makes sense, and we have in fact previously published a paper on the suitability of the CIE paradigm as an individual-differences measure (McIlhiney et al., 2022). The findings of that paper suggested that individual differences in performance on the CIE paradigm had a significant degree of stability over two timepoints (one month apart), especially when considering the correlation between CIE latent variables at each timepoint (*r* = .83). However, it should be noted that the observed-score correlation between timepoints was lower (*ICC* = .47), which was likely due to the average internal-consistency reliability found for the CIE measures. Indeed, we have already discussed issues with the CIE paradigm’s internal consistency in the Discussion (p.29). Nevertheless, based on the reviewer’s current point, we have added discussion of the CIE paradigm’s suitability as an individual-differences measure, based on evidence provided by McIlhiney et al. (2022).

Reference:

McIlhiney, P., Gignac, G. E., Weinborn, M., & Ecker, U. K. (2022). Sensitivity to misinformation retractions in the continued influence paradigm: Evidence for stability. *Quarterly Journal of Experimental Psychology*, *75*(7), 1259-1271. https://doi.org/10.1177/17470218211048986

IN-TEXT-EDIT EXAMPLE (p. 29): “In previous individual-differences research using the CIE paradigm (e.g., 17-18,32) the reported estimates of internal-consistency reliability have been problematic. Brydges et al. (17) and Sanderson et al. (18) reported estimates of α = .65 and α = .46, respectively. Similarly, McIlhiney et al. (32), who used two parallel CIE tasks in a test-retest format, reported estimates of ωtime1 = .53 and ωtime2 = .60. While these findings could suggest that the CIE paradigm suffers from similar issues found when using other experimental tasks in individual-differences research (e.g., 74), McIlhiney et al.’s (32) findings suggested that the CIE paradigm showed acceptable stability in individual-differences variation. Following recommendations cited by McIlhiney et al., we attempted to alleviate reliability issues by incorporating additional retraction and control items; however, our estimated internal consistency of ω = .57 indicated no improvement to reliability.”

4. The same issue could arise for the flanker task (a classic experimental task) and even the Stroop task (although the Stroop is widely used as an ID measure it is does seem to a strongly accentuate between groups variation and attenuate inter-individual variation). I do wonder whether the problem with the inhibition latent variable could possibly be related to the suitability of some of these tasks as ID measures.

RESPONSE: We agree with the point being made here and have updated the manuscript with a discussion of this point.

IN-TEXT-EDIT EXAMPLE (p.27): “In fact, it is even debated whether inhibition can be measured as a unitary construct, with some research suggesting that inhibition may be composed of separable but related subfactors.(65-68) However, attempts to measure these inhibition subfactors have also yielded inconsistent outcomes, as demonstrated with prepotent-response inhibition in the current study and other studies using Miyake’s EF model.(see 29 & 31 for reviews) Friedman and Miyake (31) indeed stated that many failures to replicate their EF model have resulted from issues with the (prepotent-response) inhibition factor. Perhaps a potential reason why an inhibition factor was not found in the current study, and previous studies, lies in the measures used; more specifically, evidence suggests that experimental tasks designed to create a between-subjects effect (e.g., the Stroop effect in the Stroop task) can be unreliable at producing individual differences (e.g., 69). Therefore, future studies investigating the relationship between inhibition and CIE susceptibility should carefully consider how inhibition is measured, consult past EF research that used a latent-variable approach, and select appropriate measures for forming an inhibition factor.”

5. Finally, a pretty minor but ‘big picture’ comment: it seems to me that it’s a bit of a stretch to argue that longer term, real world CIE effects, like the 5G example given in the introduction, are a ‘simple’ matter of poor WM/EF abilities (whether updating or inhibition issues). Unlike lab-based CIE tasks, in these real world situations it can be some time before disconfirming information comes to light. There seems to be an implication that the ‘action’ occurs at the point at which disconfirming information becomes available. But what if this doesn’t happen for weeks or months? Is the mental model ‘frozen’ until new information arrives? I’d expect not. I don’t think we can simply assume that people’s mental models are stable over longer periods of time – indeed, I think it’s quite possible (via confirmation bias type processes) that people seek out ‘confirming’ information and/or develop model-preserving strategies that can make updating or inhibition decreasingly likely to be effective in changing a person’s mental model (or selecting the better one). So the question is, how big a sway do WM/EF abilities have over belief change in these longer term, real world situations compared to lab-type CIE scenarios? Are they still important or do they become decreasingly important? I suspect WM/EF abilities also play a role in moderating the role of confirmation bias processes and the degree to which people adopt a ‘monological belief system’ about conspiracy theories and other potentially problematic beliefs. Importantly, I don’t think this is a huge problem for the paper – it’s fine to examine a ‘short-term CIE’. What we’ve learnt from the current paper is valuable – I just don’t think it necessarily can explain the kind of example used at the beginning of the paper.

RESPONSE: We agree that the example provided in the beginning of our paper does not map neatly onto the types of misinformation used in our paper, and have thus changed the opening example to something more suitable. Furthermore, to address the raised issue of difference in timescale between lab and real-world CIEs, we have added clarification in the Discussion that executive-function variation is likely to play a small role in real-world CIE occurrences.

IN-TEXT-EDIT EXAMPLE (p.3): “During the Australian “Black Summer” wildfires in 2019/2020, some groups repeatedly claimed that arson caused the fires; while these claims were later debunked, some continued to falsely believe in arson’s causative role, contributing to further polarisation in the climate-change debate (1-2). Such a phenomenon is a real-world example of the continued influence effect (CIE).(4) Specifically, the CIE constitutes the continued use of information in inferential reasoning after said information has been retracted or corrected. Psychological lab studies have demonstrated that the CIE can occur even when materials are fictional, suggesting that cognitive factors play a significant role in the CIE.”

(p.30): “In practical terms, the results of the current study could be useful to inform attempts to address the spread of misinformation, such as the intervention strategies summarised in The Debunking Handbook 2020.(79) While information-focused interventions have demonstrated efficacy in the lab, the efficacy of such interventions should be tested on those with lower or compromised cognitive abilities (e.g., those with compromised EF). However, it should be acknowledged that individual differences in executive function will only play a small role in real-world CIE examples. Furthermore, given that cognitive abilities can be difficult to change, intervention strategies focused on inoculating and educating against misinformation influence may help to support information-focused interventions—particularly given their generally demonstrated efficacy.(e.g.,80-91)”
